# Sensory Axonopathy Associated With Vitamin E Deficiency

**DOI:** 10.7759/cureus.13389

**Published:** 2021-02-17

**Authors:** Kok Hoe Chan, Michael O'Sullivan, Iyad Farouji, Gowthami Are, Jihad Slim

**Affiliations:** 1 Internal Medicine, Saint Michael's Medical Center, Newark, USA; 2 Internal Medicine, University of New England College of Osteopathic Medicine, Maine, USA; 3 Infectious Diseases, Saint Michael's Medical Center, Newark, USA

**Keywords:** vitamin e deficiency, sensory disorder, neuralgia, case report

## Abstract

Vitamin E deficiency can be observed in patients with malabsorption syndromes or inherited diseases such as ataxia. It is unusual for it to be a result of dietary insufficiency due to its presence in a wide variety of foods. Patients with vitamin E deficiency can present with neuromuscular disorders such as ataxia, hyporeflexia, spinocerebellar syndrome, as well as loss of vibration and proprioceptive sensation. Herein, we are presenting a case in which a previously healthy adult with no family history of genetic defects and malabsorption syndrome presented with a characteristic sensory axonopathy associated with vitamin E deficiency without any evidence of fat malabsorption. Patient reported a markedly improvement of symptoms after three-month supplementation of vitamin E. The unique part of this case was that the patient presented with neuropathic pain associated with vitamin E deficiency without any family history of inherited deficiency or any malabsorption syndrome.

## Introduction

Vitamin E in humans acts as a free radical scavenger and fat-soluble antioxidant. The active form, α-tocopherol, prevents the formation of toxic free radicals by protecting cellular membranes from oxidative stress, and inhibits the peroxidation of polyunsaturated fatty acids of membrane phospholipids [1]. Unlike other vitamin deficiencies, vitamin E deficiency is essentially never the consequence of a dietary deficiency due to its ubiquitous distribution in a wide variety of food. Vitamin E deficiency has been linked to malabsorption syndromes which can be due to chronic cholestasis, pancreatic insufficiency or intestinal malabsorption. Inherited genetic defects such as α-TTP (ataxia with vitamin E deficiency [AVED]), apolipoprotein B (homozygous hypobetalipoproteinemia), or in the microsomal triglyceride transfer protein (abetalipoproteinemia) have been linked to vitamin E deficiency [2]. Patients with a genetic defect typically develop neurological symptoms by their first or second decade, whereas the development of symptoms in those with acquired fat malabsorption syndromes takes multiple decades [3]. Herein, we describe a case in which a previously healthy adult with no family history of genetic defects and malabsorption syndrome presented with a characteristic sensory axonopathy associated with vitamin E deficiency without any evidence of fat malabsorption.

## Case presentation

A 45-year-old gentleman, originally from Haiti, immigrated to the United States in 1999, with no past medical history presented to the emergency department with abnormal sensation with numbness and tingling over bilateral lower extremities. Symptoms began two days prior and were initially localized to the right lower extremity. Symptoms progressed to involve the distal left lower extremity and spread proximally to the pelvis. He noted subjective left greater than right lower extremity weakness. He denied low back pain, incontinence of bowel or bladder, or muscle fasciculation, disruption of balance, gait, perceived upper extremity weakness, visual changes, or paresthesias. No trauma at the back or leg has been reported and it was the first-time patient experience these symptoms. There was no headache, hearing nor visual difficulties, no abnormal sensation over the upper extremities. There were no constitutional symptoms such as fever, chills, night sweats, anorexia, or weight loss. Patient has no history of recent travel, tick bite, rash, flu-like symptoms or diarrhea. He is single, heterosexual, currently not sexually active and no history of sexually transmitted diseases. He has no known drug allergies. He denies tobacco, alcohol, recreational drug use and over-the-counter prescriptions or medications. 

Triage vital signs were normal, including: temperature 99.8°F, blood pressure 118/78 mmHg, heart rate 72/min, respiratory rate 14/min and oxygen saturation was 98% on room air. Cranial nerves were grossly intact, there were no limitations in the upward gaze or visual field constriction. Neurological examination showed hypertonicity in bilateral lower extremities with hyperesthesia over the T7-T8 regions and bilateral lower extremities. Muscle strength was 5/5 on the left lower extremity and 4+ on the right lower extremity. Reflexes on the knee and ankle on the right side were 3+. There was normal vibration sense and joint position sense bilaterally. There was a normal extensor plantar response. Gait was limping and no pronator drift noted. No truncal or limb ataxia was present. Other physical examination including head, neck, spine, lung, heart, abdomen and extremities were unremarkable. 

**Figure 1:**
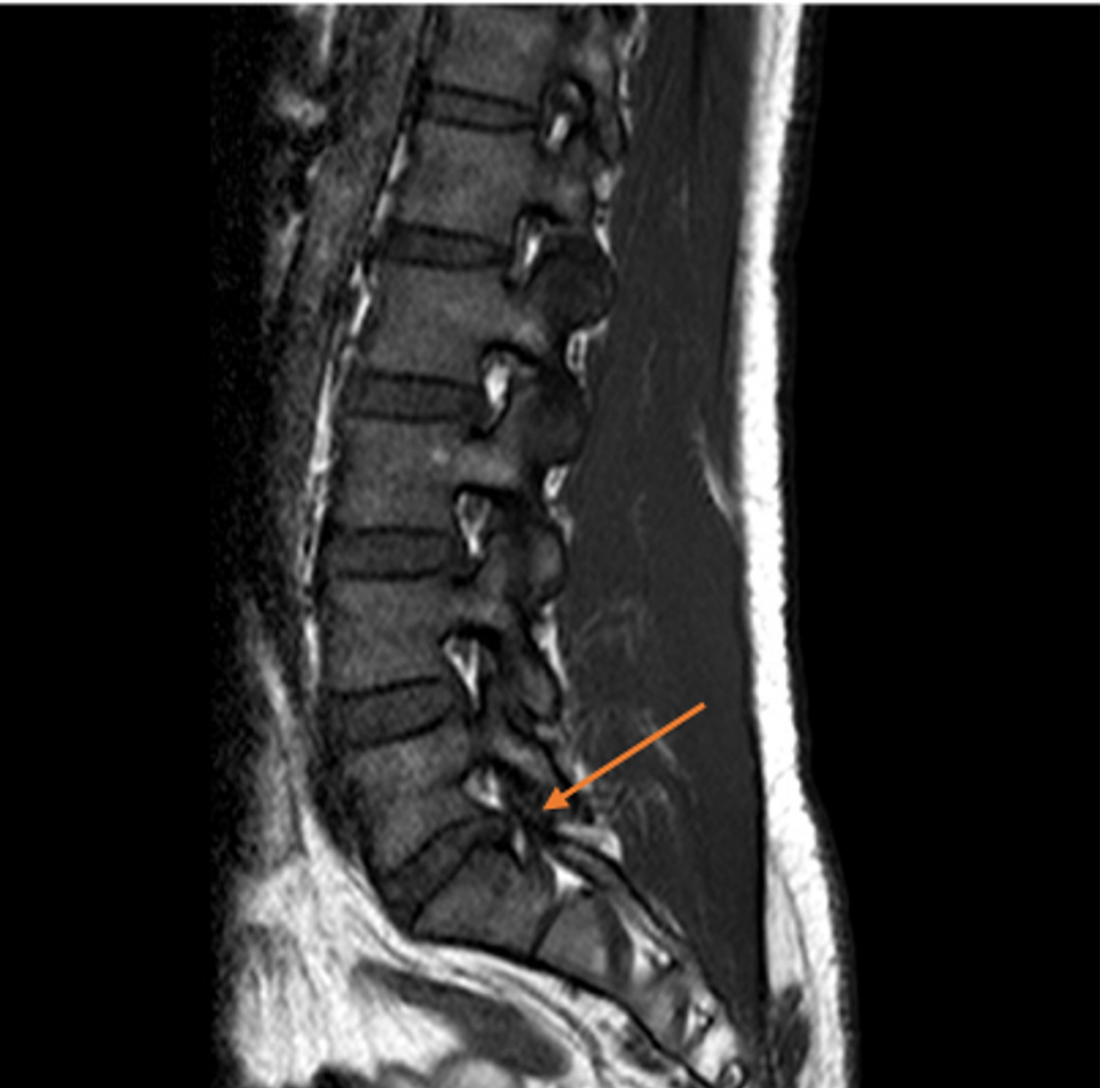
MRI of the lumbar spine showed degenerative retrolisthesis of L5 on S1 with a degenerative L5-S1 disk cartilage and with a small left-sided disk protrusion (sagittal view).

**Figure 2:**
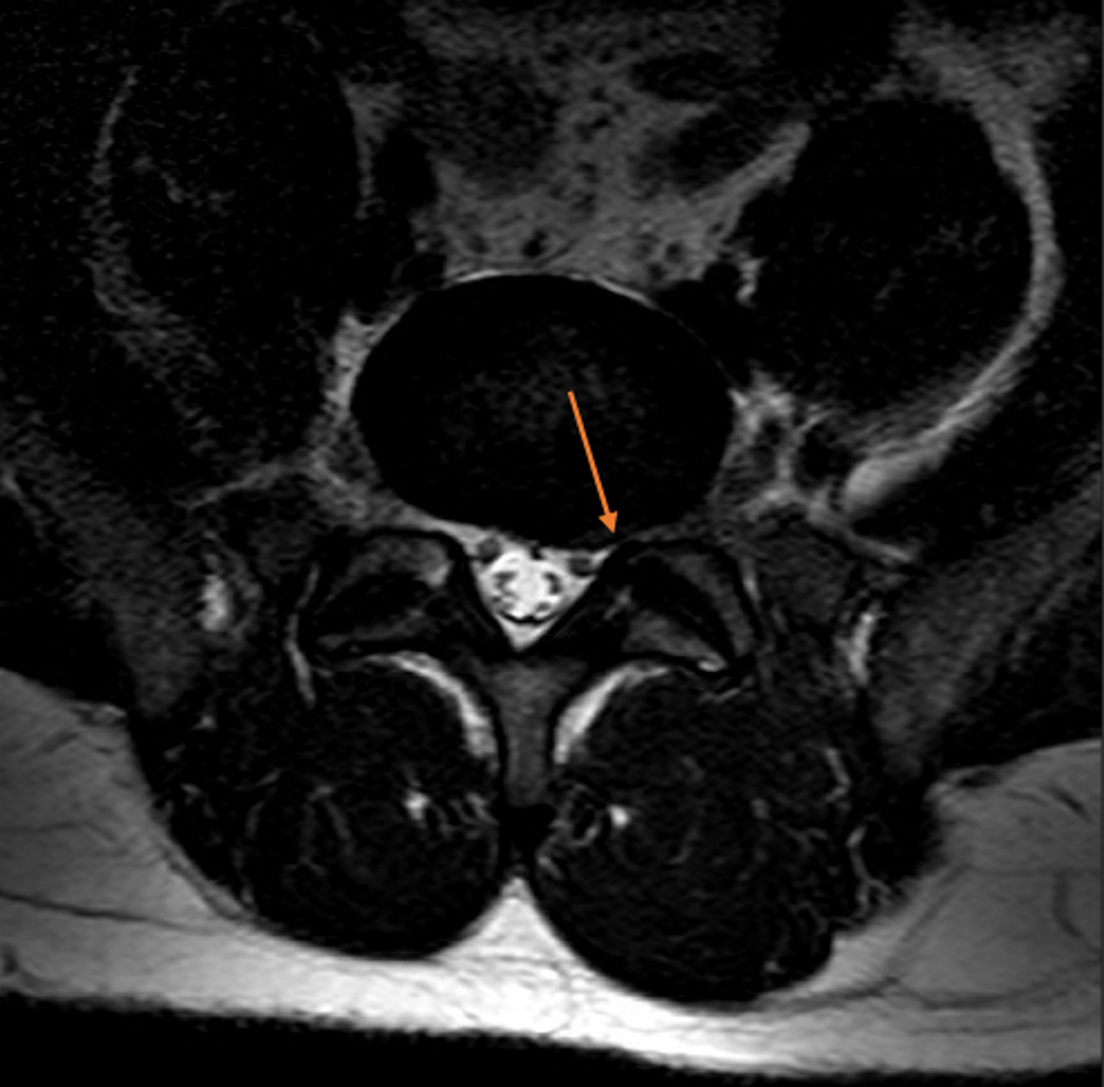
MRI of the lumbar spine showed degenerative retrolisthesis of L5 on S1 with a degenerative L5-S1 disk cartilage and with a small left-sided disk protrusion (axial view).

Further investigation confirmed evidence of vitamin E deficiency. The concentration of alpha-tocopherol was measured at <5.8 mg/L (normal range: 7.0-21.5 mg/L). The vitamin E/cholesterol ratio was 0.03 mg/mg suggesting pure vitamin E deficiency. Total creatinine kinase (CK) was 556 U/L (normal range: 52-336 U/L). Other laboratory investigations confirmed a normal full blood count, glucose, liver, kidney function, fasting lipids, thyroid function tests, Vitamin B12, folic and copper. He also had a negative HIV, Hepatitis B and C, RPR and ANA on the chemistry panel. Cerebrospinal fluid (CSF) was performed which showed normal protein, white cell counts, red blood cells count, glucose and opening pressure. CSF also showed a negative VDRL, angiotensin-converting exam, West Nile Virus, HSV, Borrelia Burgdorferi antibodies, oligoclonal bands, and normal IgG index. MRI of the brain confirmed normal intracranial appearances including no cerebellar atrophy. MRI of the thoracic spine was unremarkable, and MRI of the lumbar spine showed degenerative retrolisthesis of L5 on S1 with a degenerative L5-S1 disk cartilage and with a small left-sided disk protrusion without descending or exiting nerve root compression (Figures 1, 2). 

Patient was diagnosed with sensory axonopathy secondary to vitamin E deficiency. Physical examination also did not show significant muscle weakness and areflexia/hyporeflexia. Other CSF analysis and chemistry panel were normal. Patient was advised to take high-dose vitamin E in the form of D-alpha tocopherol supplementation at 800 IU/day. Patient has a loss of follow-up after the initial presentation but on a phone call at three months after vitamin E supplement, patient admits a markedly improvement in the symptoms and gait.

## Discussion

Vitamin E deficiency is rare in humans due to the wide variety of tocopherols in most diets. Even vegetarian and vegan diets are replete with sources of vitamin E, but some severe protein-deficient diets can cause a vitamin E deficiency [4]. The main cause of vitamin E deficiency is from fat malabsorption, which includes conditions such as cystic fibrosis, cholestatic liver disease, and small bowel resection [5,6]. There are also certain genetic disorders that can predispose one to vitamin E deficiency. In a United States national survey, the 5th percentile for vitamin E serum levels was 0.62 mg/dl, while the 25th percentile was 0.79 mg/dl [7].

A deficiency in vitamin E can result in neuromuscular disorders and hemolysis. Low serum levels are defined as below 0.5 mg/dl and often cause no appreciable symptoms. When symptoms do appear, they usually present as a neuropathy or myopathy. The neuropathy consists of a spinocerebellar syndrome with variable peripheral nerve involvement [8]. Clinical manifestations include ataxia, hyporeflexia, and decreased proprioceptive or vibratory sensation. Early vitamin E deficiency typically has hyporeflexia, with advanced illness progressing to areflexia, Other late findings include dysphagia and dysarthria, cardiac arrhythmias, ophthalmoplegia, and possible blindness [8]. Myopathic conditions stemming from vitamin E deficiency include intestinal lipofuscinosis which is a brown pigmentation of bowel that occasionally presents with bowel dilation and pseudo-obstruction [9]. This is a rare condition that is thought to occur from the buildup of lipofuscin in smooth muscle mitochondria. It is interesting to note that our patient had a pronounced limp, but no obvious ataxia, his vibratory sensation was intact, as were his lower extremity reflexes, which suggest that this patient had relatively early vitamin E deficiency. Premature infants with vitamin E deficiency can experience a hemolytic anemia, as vitamin E deficiency shortens the life span of red blood cells [10].

The current recommendations for screening of vitamin E deficiency are for patients with the conditions that are known to cause a deficiency in the vitamin, listed above. Other reasons for screening include patients with a family history of vitamin E deficiency, as well as those suffering from unexplained spinocerebellar neuropathy or ataxia [11,12]. As our case shows, neuropathic symptoms outside of spinocerebellar neuropathy can present in patients with vitamin E deficiency. Patients with other neuropathic symptoms should be included in screening as well. In addition to that, there are some new studies that suggest the possibility to use vitamin E in treating the neuropathic pain [13].

Treatment for vitamin E deficiency includes large oral doses of vitamin E [14]. For adults with vitamin E deficiency due to fat malabsorption, doses typically start at 50-500 mg/day, then adjusted to achieve normal serum measurements of alpha tocopherols. Patients with severe cholestatic disease or genetic disorders that interfere with vitamin E transport may not respond to even high doses of alpha-tocopherol [15]. Intramuscular vitamin E is effective but is often not widely available and is somewhat impractical as it requires frequent dosing.

This case demonstrates an example of isolated Vitamin E deficiency in a patient with no genetic predisposition or history of malabsorption syndromes. As mentioned previously, the signs and symptoms of vitamin E deficiency presented in this patient outside of the recorded age range that is expected in any genetic condition. Inadequate Vitamin E intake was excluded as the patient reported a normal dietary intake. Fat malabsorption was excluded based on clinical grounds and laboratory tests. There is a condition known as ataxia with vitamin E deficiency (AVED) which is inherited in an autosomal recessive pattern [16]. The condition is characterized by progressive cerebellar ataxia similar in presentation to Friedrich’s ataxia. Given the concomitant presence of specific neurological symptoms and a low vitamin E level, this inherited condition could not be ruled out. However, the patient presented specifically with neuropathic pain rather than ataxia. Furthermore, the patient was out of the typical age range in which a genetic disorder of vitamin E metabolism is diagnosed. In either case, further genetic testing may prove helpful in understanding the pathogenesis of this patient’s condition, but it will not change the method of treatment with vitamin E supplementation. 

## Conclusions

In conclusion, this case highlights the variable presentation of vitamin E deficiency. It is capable of mimicking a number of other conditions, and oftentimes there is no clear pathogenesis behind it. In this case, the patient’s primary symptom was sensory neuropathy; however, the most common symptom of spinocerebellar ataxia was absent. The patient also lies outside of the age range that genetic disorders of vitamin E metabolism usually present, and he did not display evidence of malabsorption. This indicates that there may still be unexplained mechanisms behind vitamin E deficiency. A more in-depth understanding of vitamin E metabolism and the pathogenesis behind vitamin E deficiency may lead to better diagnostic tools and earlier detection in at-risk patients, thus improving morbidity in the condition.
